# Higher-Order Chromatin Structures of Chromosomally Integrated HHV-6A Predict Integration Sites

**DOI:** 10.3389/fcimb.2021.612656

**Published:** 2021-02-26

**Authors:** Michael Mariani, Cosima Zimmerman, Princess Rodriguez, Ellie Hasenohr, Giulia Aimola, Diana Lea Gerrard, Alyssa Richman, Andrea Dest, Louis Flamand, Benedikt Kaufer, Seth Frietze

**Affiliations:** ^1^ Department of Biomedical and Health Sciences, College of Nursing and Health Sciences, University of Vermont, Burlington, VT, United States; ^2^ Institute of Virology, Freie Universität Berlin, Berlin, Germany; ^3^ Division of Infectious Disease and Immunity, CHU de Québec Research Center-Université Laval, Quebec City, QC, Canada; ^4^ University of Vermont Cancer Center, Burlington, VT, United States

**Keywords:** epigenetics, chromatin 3D architecture, latency, herpesvirus (hhv-6), gene expression

## Abstract

Human herpesvirus -6A and 6B (HHV-6A/B) can integrate their genomes into the telomeres of human chromosomes. Viral integration can occur in several cell types, including germinal cells, resulting in individuals that harbor the viral genome in every cell of their body. The integrated genome is efficiently silenced but can sporadically reactivate resulting in various clinical symptoms. To date, the integration mechanism and the subsequent silencing of HHV-6A/B genes remains poorly understood. Here we investigate the genome-wide chromatin contacts of the integrated HHV-6A in latently-infected cells. We show that HHV-6A becomes transcriptionally silent upon infection of these cells over the course of seven days. In addition, we established an HHV-6–specific 4C-seq approach, revealing that the HHV-6A 3D interactome is associated with quiescent chromatin states in cells harboring integrated virus. Furthermore, we observed that the majority of virus chromatin interactions occur toward the distal ends of specific human chromosomes. Exploiting this finding, we established a 4C-seq method that accurately detects the chromosomal integration sites. We further implement long-read minION sequencing in the 4C-seq assay and developed a method to identify HHV-6A/B integration sites in clinical samples.

## Introduction

Human herpesvirus 6A (HHV-6A) and 6B (HHV-6B) are two closely related and ubiquitous betaherpesviruses (Zerr et al., 2005). Most people are infected with HHV-6B as infants, while the etiology of HHV-6A remain poorly defined (Okuno et al., 1989; De Bolle et al., 2005). Following primary infection, HHV-6A/B establishes latency in the host for life (Flamand, 2018). During this latent state, viral gene expression and viral load are not detectable (Saviola et al., 2019). However, the virus can sporadically reactivate in the host, resulting in the production of infectious virions and viral transmission (Endo et al., 2014). HHV-6A/B reactivation has been linked to a variety of pathologies including encephalitis, graft rejection and a spectrum of other diseases (Aimola et al., 2020).

HHV-6A/B are unique among human herpesviruses as they are able to integrate their genomes into the telomeres of human chromosomes (Arbuckle et al., 2010; Osterrieder et al., 2014). Chromosomal integration of HHV-6A/B occurs very efficient *in vitro* and is dependent on telomere sequence arrays present at the ends of the virus genome (Wallaschek et al., 2016; Gravel et al., 2017). HHV-6A/B can also integrate into the germline, resulting in individuals harboring the virus in every nucleated cell of the body and inherit it to their offspring. This inherited chromosomally-integrated HHV-6 (iciHHV-6) is present in about 1% of the human population (Kaufer and Flamand, 2014) with several independent integration events occurring thousands of years ago (Aswad et al., 2020). Integration into the telomeres allows maintenance of the virus genome in iciHHV-6 individuals and latency (Arbuckle et al., 2010); however, it remains unknown if integration into the telomeres contributes to the silencing of the virus genome.

Here, we explored if, how and when the HHV-6A genome is silenced upon integration using an *in vitro* integration model and RNA-sequencing. We demonstrate that HHV-6A genes are silenced upon integration over the course of 7 days. We employ circular chromosome conformation capture assays (4C-seq) to assess the higher order chromatin structures formed between the virus and host genome in these cells. Because HHV-6A/B integrate into highly repetitive telomeric regions of individual human chromosomes, we used 4C-seq-based analysis as a tool to identify integration sites. Our 4C-seq approach employs novel scoring method as well as Oxford Nanopore minION long-read sequencing to effectively identify integration sites in human telomeres. Overall, this study provides a better understanding of the chromatin programs that may regulate HHV-6A latency and provide novel diagnostic methods to determine chromosomal integration sites.

## Materials and Methods

### Ethics

Specimens were obtained from the Fred Hutch Research Cell Bank, which prospectively collects and cryopreserves peripheral blood mononuclear cells (PBMCs) from donors and recipients. The University of Washington Institutional Review Board approved use of the iciHHV-6 specimens from the Fred Hutchinson Cancer Research Center and use of anonymized excess HHV-6-positive samples submitted for testing at the University of Washington Virology lab.

### Cell Lines and Virus

SMC cells harboring integrated HHV-6A virus (iciHHV-6A cells) were immortalized following transduction with a lentiviral vector (pLenti CMV/TO SV40 small + large T (W612-1), a gift form Eric Campeau and obtained from Addgene (plasmid #22298)) and expressing the SV40 T antigens. SMCs were cultured in Dulbecco’s modified Eagle’s medium (DMEM) supplemented with 20% fetal bovine serum (FBS), 1% penicillin streptomycin (Pen/Strep), and 1% L-Glutamine. Human epithelial kidney 293 T (293 T, ATCC CRL-11268) cells were cultured in the same medium but supplemented with 10% FBS. All cells were maintained in 10 cm^2^ flasks as a monolayer culture in a humidified 5% CO2 air incubator at 37°C. Bacterial artificial chromosome (BAC)-derived HHV6A (strain U1102) expressing green fluorescent protein (GFP) under the control of the HCMV major immediate early (IE) promoter (293-HHV-6A virus) was propagated in JJHan cells as described previously (Tang et al., 2010b). 293 T cells were infected with 293-HHV-6A virus and GFP positive cells were isolated using a FACS AriaIII cell sorter (BD Biosciences). Clones harboring the integrated 293-HHV-6A genome (ciHHV-6A cells) were identified by quantitative PCR (qPCR) and HHV-6A integration was confirmed by fluorescent *in situ* hybridization (FISH). iciHHV-6B+ human lymphocytes (NCBI GenBank: KY315552) were isolated from an infected patient as described by Aswad et al. (Aimola et al., 2020; Aswad et al., 2020).

### Fluorescence *In Situ* Hybridization

FISH was performed with digoxigenin-labeled bacterial artificial chromosome (BAC) probes against both the HHV-6A genome and individual chromosomal reads essentially as described previously (Kaufer et al., 2011; Kaufer, 2013). Slides were mounted using DAPI Vectashield (Vector Laboratories) and images were taken with an Axio Imager M1 (Zeiss).

### RNA-Seq Analysis

RNA was extracted from an HHV-6A infected 293T cell line using Trizol Reagent (Life Technologies) and purified using Direct-zol RNA MicroPrep Kit (Zymo Research #R2060) following the manufacturer’s instructions. One microgram of total RNA was depleted of ribosomal RNA (rRNA) using the KAPA RiboErase Kit (#KR1142) and libraries were prepared using the KAPA Stranded RNA-seq Library Preparation Kit (#KR0934). Libraries were quantified using Qubit (Life Technologies), and quality was assessed using the Agilent Bioanalyzer High-Sensitivity DNA kit (Agilent Technologies). Barcoded libraries were pooled and sequenced on an Illumina HiSeq 2000 to obtain 100-bp paired-end reads. RNA-seq data were processed using STAR as described previously (Dobin et al., 2013) using human (hg38) and a custom HHV-6A reference genomes (NC_001664.4) modified to contain GFP transgenes. BAM files were sorted and converted to SAM files using SAMtools (Li et al., 2009) and reads were counted with featureCounts (Liao et al., 2014) against the corresponding viral GTF file and normalized in DESeq2 (Love et al., 2014). Alignment of viral reads across the HHV-6A genome was visualized using the “seqsetvis” R library (Boyd, 2020) and in-house scripts.

### 4C-Seq Library Preparation and Sequencing

In order to identify the higher order chromatin structure and virus-host DNA contacts, 4C-seq libraries were prepared using both ciHHV-6A and iciHHV-6A samples as described previously (Krijger et al., 2020), using HindIII/DpnII restriction enzymes. Cells were cultured as previously described (Saviola et al., 2019) and fixed with 1% formaldehyde prior to being snap frozen. For each 4C library, ten million cells were used unless otherwise noted. Inverse PCR was performed in 50 µl reactions using 200 ng 3C library template, 25 µl Q5 Hot Start High-Fidelity DNA Polymerase 2X Mastermix (New England Biolabs), 1.5 µl of 10uM forward and reverse inverse PCR primers and water under the following conditions: 30 s 98°C, 10 s 98°C, 30 s 55°C, 2 m 72°C, 5 m 72°C for a total of 25 cycles. PCR products were cleaned up using the PCR purification kit (Thermofisher Scientific) using the B3 reagent to exclude fragments less than 300 base pairs or with Ampure XP beads (Beckman Coulter) and eluted in water. Library indexing was performed using the DNA HT Dual Index Kit (Illumina) or with custom dual indexing primers with an additional eight cycles of PCR. Indexing of nanopore libraries was performed with custom 12-nucleotide dual index primers with eight additional PCR cycles. All primers sequences are listed in **Supplementary Table 1**. Libraries were pooled and sequenced with either HiSeq2000 or MinION platforms. The experiments were performed with samples from separate cell culture dates. For the Illumina viewpoint comparison libraries, two replicates were performed for two separate viewpoints. For the ONP titration and viewpoint comparison studies, one and two replicates were performed, respectively.

### 4C-Seq Primer Design

We developed a 4C-seq primer design tool^1^ to facilitate the generation of the inverse PCR primers necessary for 4C-Seq assays in a genome-agnostic manner for use with experiments involving custom genomes (i.e., chromosomally integrated HHV-6A). A fasta file containing the HHV-6A genome (NC_001664) was obtained from NCBI and uploaded into the design tool, selecting HindIII and DpnII restriction endonucleases, and minimum fragment sizes 500 bp for the first fragment and 300 bp for the second fragment were used (van de Werken et al., 2012).

### 4C-Seq Analysis

4C-seq *trans* interactions were determined using a “window” method as described by Kim et al., (2020). Briefly, the virus viewpoint regions at the beginning of the reads were trimmed off using Cutadapt (Martin, 2011) software and the remaining human portions were aligned to the human UCSC hg38 reference genome using bowtie2 (version 2.3.5) (Langmead et al., 2009; Langmead and Salzberg, 2012). P-values for each mapped base were then calculated for each 10kb window across all chromosomes using a Poisson formula. Final, significant peak regions are then called using the MACS2 (version 2.2.7) bdgpeakcall function (Zhang et al., 2008). Significant Interacting *trans* peak regions were identified using the above window method for each replicate, for each viewpoint, in both the ciHHV-6A and iciHHV-6A samples. These regions were plotted as circos plots using the Circlize R library (Gu et al., 2014). Bedtools (Quinlan and Hall, 2010) was used to find the intersection of the *trans* regions and the 127 epigenomes 25-state imputation based chromatin state model created with ChromImpute (Ernst and Kellis, 2012; Ernst and Kellis, 2015) and made available from the Wang Lab^2^ (Jin Wook Lee). For ciHHV-6A cells, chromatin state annotation was also performed using fetal kidney and fetal adrenal gland tissues – also made available from the Wang lab^2^. 4C *trans* peaks and signals were also compared to signals for the repressive histone marks H3K9me3, NCBI GEO: GSE66530 (Hattori et al., 2016), and H3K27me3, NCBI GEO: (Lamb et al., 2019), in HEK293 cells and compared to the same marks, NCBI GEO: GSE121984, in iciHHV-6A cells (Saviola et al., 2019).

4C-seq *cis* interactions were determined using the program peakC (Geeven et al., 2018). Telomere content comparison was assessed by TelomereHunter (Feuerbach et al., 2019). Illumina data was simulated using ART (Huang et al., 2012) set to HiSeq2500 data. Reads were produced to cover the entire UCSC hg38 reference genome at 1X coverage. Then, a number of simulated reads equivalent to the number of reads sequenced between 4C data were randomly sampled from the simulated data. TelomereHunter was run on all samples and a non-parametric Wilcoxon test was performed to compare 4C-seq reads to simulated reads.

To identify the chromosomes harboring HHV-6A integration, a scoring system was developed and coded using the R programming language. Accordingly, trimmed reads were aligned to the hg38 genome using bowtie2, or BWA-MEM (Li, 2013) for MinION reads and each read that maps within 500 kb of any autosome terminus was scored. The total scores for reads within these 500 kb regions are tallied and then compared to each other in one of two ways. If replicates are available, a two-way ANOVA is first performed with chromosome end sum scores as the outcome variable and individual chromosome ends as the categorical predictor variable. TukeyHSD pairwise-*post hoc* tests are then performed and the chromosome end with the highest mean -log_10_ p-value is determined to be the most likely candidate. A significance of 0.05 was used for this approach. If replicates were not available for a specific viewpoint/cell type combination, the chromosome end scores were all compared to each other using Wilcoxon nonparametric tests. The chromosomal end with the largest -log_10_ p-value adjusted for multiple test comparison was chosen as the most likely candidate and an alpha of 0.05 was used as a cutoff of significance. Furthermore, statistical ranking *via* the aforementioned pairwise post-hoc tests from the algorithm score sums (for each chromosome end) to identify the ends of each chromosome as most significant candidate for HHV-6A integration.

## Results

### Dynamics of HHV-6A Gene Expression During the Establishment of Latency

**Figure 1:**
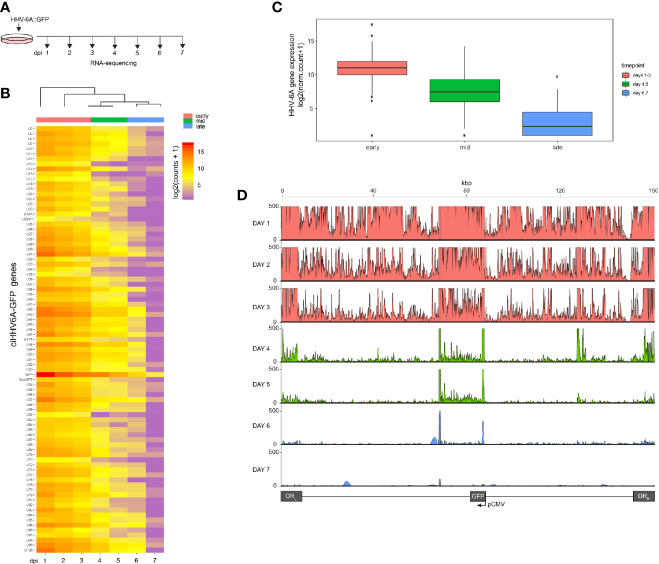
RNA-Seq time course reveals the establishment of transcriptionally silent state upon human herpesvirus 6A (HHV6A) infection. **(A)** 293T cells were infected with ciHHV-6A and green fluorescent protein (GFP) expressing cells were isolated by FACS. RNA-seq analysis was performed on the cells during the integration phase at the indicated days post infection (dpi). **(B)** Cluster analysis of RNA-seq expression of HHV-6A GFP genes, showing expression values of normalized counts with a pseudo-count of one. **(C)** Boxplots of mean viral gene expression shown for each time point group. **(D)** RNA-seq signal plotted across the HHV-6A genome for each day. Viral expression is almost completely absent by day 7 indicating the establishment of a transcriptionally silent latent infection. At the later timepoints (e.g., days 4-6), two prominent peaks can be seen flanking the U41 and U42 genes, the origin of lytic DNA replication (oriLyt) and the CMV promoter and GFP regions.

We recently reported that the chromosomally integrated HHV-6A genome exists in a compacted transcriptionally silent state (Saviola et al., 2019). To study the kinetics of HHV-6A gene silencing upon HHV-6A infection, we performed a 7-day time course in human 293T cells. 293T cells are susceptible to HHV-6A infection and were previously established as a model for studying virus integration (Arbuckle et al., 2010; Gravel et al., 2017; Saviola et al., 2019). Cells were infected with recombinant HHV-6A virus expressing GFP under the control of the major immediate-early (IE) HCMV promoter (Tang et al., 2010a; Wallaschek et al., 2016). Infected GFP-positive cells were isolated by FACS sorting 16 h post infection and subsequently cultured. The presence of the HHV-6A genome was confirmed by qPCR and integration was validated by FISH at day seven post-infection. Infected cells were collected daily until 7 days post-infection (dpi) and processed for gene expression profiling *via* RNA-sequencing (**Figure 1A**). The expression of all HHV-6A genes progressively decreases over the 7-day period as visualized *via* a clustered heatmap (**Figure 1B**). The observed gene silencing over this period course can be grouped into three clusters based on expression levels of all viral genes (early, mid and late). The collective expression of genes is reduced over a 3-day period, followed by near complete silencing from 5–7 dpi (**Figure 1C**). Thus, HHV-6A gene expression upon infection is silenced within 7 days in 293T cells harboring the integrated virus genome.

### Higher-Order Chromatin Interactions of Chromosomally Integrated HHV-6A

**Figure 2:**
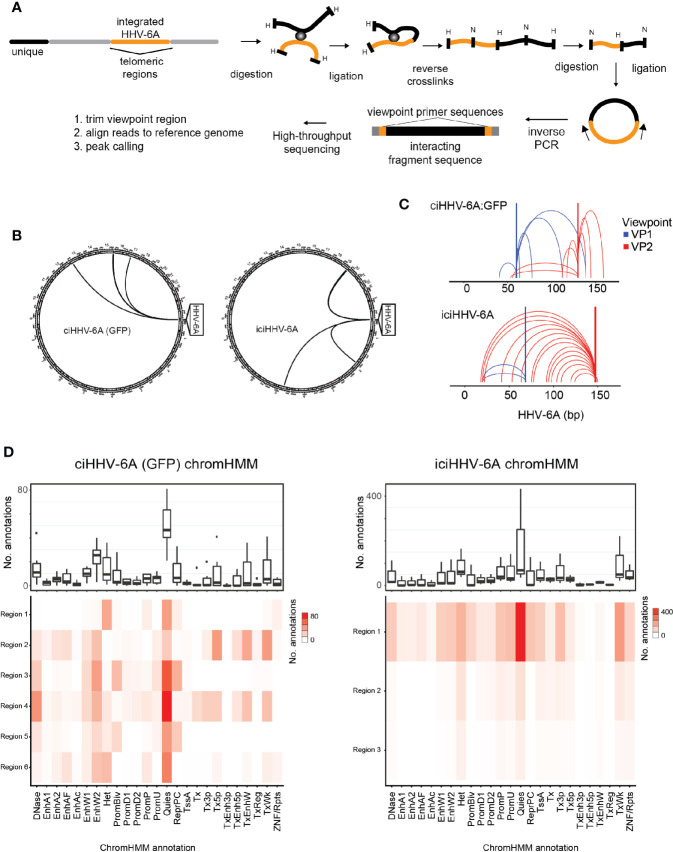
Chromatin interactions of chromosomally integrated human herpesvirus 6A (HHV-6A). Circular Chromosome Conformation Capture (4C-seq) assay was employed using chromosomally integrated HHV-6A cell models to investigate virus-host chromatin interactions. **(A)** 4C-seq assay with ciHHV-6A cells. The integrated HHV-6A genome (yellow) is shown with its flanking telomeric regions (gray) and telomere-proximal unique portions of a human chromosome (black). Indicated are the restriction endonucleases sites used in the 4C-seq assay (HindIII and DpnII are indicated as ‘H’ and ‘D’, respectively). Inverse PCR was performed with distinct viewpoint primers and analysis interaction regions was performed as shown. **(B)** HHV-6A *trans* interactions for *in vitro* integration model (ciHHV-6A) and the chromosomally inherited iciHHV-6A cell model (iciHHV-6A). Significant interactions are visualized using circos plots both viewpoints are shown for ciHHV-6A cells (left) and for iciHHV-6A cells(right). **(C)** HHV-6A *cis* interactions identified for two separate HHV-6A viewpoints. Inverse primers were designed for two viewpoint regions chosen at ~ 64 kb and 148 kb along the HHV-6A genome. Note that the HHV6A-GFP genome is slightly larger than the canonical HHV-6A genome due to the additional presence of recombinant elements. **(D)** Host interaction regions (*trans)* are annotated using the 127 epigenomes 25-state chromatin state model database for ciHHV-6A (left) and iciHHV-6A (right). The host interaction regions contain the indicated chromatin database annotations. Displayed in boxplots (top) and as a heatmap (below).

The epigenetic mechanisms that regulate gene silencing in chromosomally integrated HHV-6A/B remain poorly characterized. We previously demonstrated that the integrated viral genome forms repressed heterochromatin domains (Saviola et al., 2019). We further hypothesized that higher-order chromatin interactions play a role in virus gene silencing. To investigate higher-order chromatin interactions between chromosomally integrated HHV-6A, we designed 4C-seq assays using distinct viewpoint regions designed against distinct HHV-6A regions (**Figure 2A**). To obtain the inverse PCR primers required for 4C analysis, we developed a genome agnostic 4C primer design tool (see methods). Two distinct viewpoints (vp1 vs. vp2) were designed based on optimal fragment length and restriction enzyme sequence along the length of the ~160 kb HHV-6A genome. The HHV-6A genomic regions targeted by viewpoint vp1 and vp2 primers are located adjacent to the U39 gene-encoding glycoprotein B (gB) (position: 64 kb) and the U95 gene (position: 148 kb), respectively (Nicholas and Martin, 1994). With this approach, we assessed the HHV-6A chromatin conformation in a previously described 293T cells line harboring the chromosomally integrated HHV-6A (ciHHV-6A) (Saviola et al., 2019) and in iciHHV-6A+ patient-derived cells (iciHHV-6A). We obtained approximately 2 hundred thousand to 2 million reads per viewpoint replicate across 2 independent 4C-seq assays, of which 85.93% of reads mapped to the combined reduced human+HHV-6A genome (hg38 + NC_001664.4 or recombinant GFP genome, depending on the sample) (**Supplementary Table 2**). Significant ‘*trans*’ interacting regions between the HHV-6A genome and the human genome were identified for both *in vitro*-derived (ciHHV-6A) and patient-derived iciHHV-6A cells (**Figure 2B**). For ciHHV-6A, we found a total of 6 high-confidence interactions that intersected between viewpoints, and a total of 3 interactions were between viewpoints for iciHHV-6A cells. In addition, a number of *cis*-interacting regions were identified for both viewpoints (**Figure 2C**; **Supplementary Table 2**). In line with other 4C-seq results (Krijger et al., 2020) there were relatively high densities of interactions that occur near and between viewpoints. Overall, there were a number of cis interactions identified in each cell types suggesting that virus genome may reside in a highly folded compartment similar to a topologically associated domain.

To assess the chromatin features of the identified interaction regions, we annotated the identified interacting human regions using available chromatin state segmentations compiled from multiple human tissue types (Ernst and Kellis, 2015). This annotation dataset is derived from 12 epigenetic features and from 127 original reference epigenomes. We found that the human chromatin that interacted with HHV-6A is largely composed of quiescent and heterochromatin states, whereas enhancer and transcriptional activation annotations occur to a lesser degree (**Figure 2D**). Similarly, chromatin state annotations derived from single representative cell types exhibit similar chromatin state patterns at the HHV-6A interacting genomic regions (**Supplementary Figure 3**). We further inspected the enrichment of the repressive histone modifications H3K9me3 and H3K27me3 at both ciHHV-6A and iciHHV-6A 4C-seq peaks using ChIP-seq data from each respective cell-line (**Supplementary Figure 2**). In both ciHHV6-A and iciHHV6-A, the majority of the significant 4C-seq peaks overlap directly with these repressive histone marks. These results indicate that the integrated HHV-6A genome forms higher order chromatin structures within the viral genome as well as with regions of the human genome that are enriched with repressive chromatin. Overall, these results provide insight into HHV-6 chromatin structures within the host cell nucleus.

### Proximity Chromatin Ligation Reveals Integration Sites

**Figure 3:**
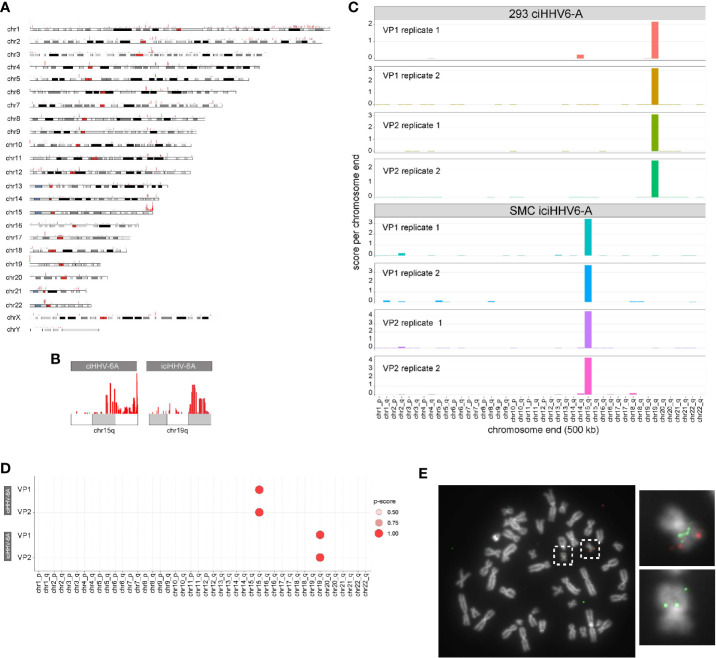
**(A)** Identification of chromosome ends harboring integrated human herpesvirus 6A (HHV-6A). **(A)** Genome-wide karyotype plot showing 4C-seq read mapping density for iciHHV-6A, viewpoint 6a10 replicate 1, across each human chromosome. **(B)** Karyotype plots showing 4C-seq read mapping density for chromosomes 15 and 19 for each cell-line. **(C)** 4C-seq signal clustering method to score all the reads within 500 kb from each chromosomal end. The sum of these scores is represented in the bar plots for each replicate for each viewpoint in both ciHHV-6A and iciHHV-6A cell types. **(D)** Statistical measure of the likelihood that a specific chromosome harbors integrated HHV-6A. For datasets that contain replicates, an ANOVA was performed using the score from our algorithm across all chromosome ends followed by pairwise Tukey’s HSD post-hoc tests. The “p-score” is the mean -log10(p-value) value for all pairwise comparisons divided by (normalized by) the maximum mean -log10(p-value). **(E)** Integration sites were confirmed *via* fluorescent *in situ* hybridization using probes complimentary to chr19q (green) and the HHV-6A genome (red) in iciHHV-6A (SMC) cells.

HHV-6A/B integration sites were first detected by FISH (Nacheva et al., 2008), and the virus-host junction of three different integrations was sequenced using a PCR-based approach. Although PCR amplification and Sanger sequencing provided sequence information, this approach requires previous knowledge of the chromosomal location of the virus genome (Huang et al., 2014; Tweedy et al., 2016) and is prone to amplification biases and sequencing errors due to the repetitive nature of the region (Arbuckle et al., 2010; Arbuckle et al., 2013; Huang et al., 2014; Ohye et al., 2014; Gulve et al., 2017). Genome-wide mapping of short read sequence data to human subtelomeres is challenging due the repetitive nature of subtelomeric regions. It is well-documented that higher order chromatin interactions largely occur in *cis* in large megabase pair (Mbp)-sized topologically associated domains (TADs) (Szabo et al., 2019). Because HHV-6A integrates into a host chromosome, the extra-chromosomal *trans* 4C-seq interactions that are identified should behave like endogenous intrachromosomal *cis* interactions, i.e., 4C-seq should show distinct interacting regions between the host and HHV-6A genomes. With this in mind, we hypothesized that 4C-seq data could identify HHV-6A integration sites, and visualization of 4C alignments to the human genome reveals particular telomeric ends that are enriched with 4C signal. **Figure 3A** shows enrichment at the distal end of chromosome 15q relative to the rest of the genome in ciHHV-6A cells. In addition, clustering of reads at the distal ends of chromosome 15q and chromosome 19q in ciHHV-6A and iciHHV-6A, respectively, can be seen in **Figure 3B**. Further, we identified significantly more telomeric sequences in our 4C-seq data relative to simulated reads of equal size and GC content (**Supplementary Figure 1**). To enable systematic analysis of HHV-6A integration sites using 4C-seq, we established a scoring procedure to assess the chance of integration across all chromosomes based on clustered read mapping (see methods). The ends of chromosome 15q and chromosome 19q rank as the highest scoring chromosome ends for ciHHV-6A and iciHHV-6A, respectively, in terms of read mapping density and integration probability (**Figures 3C, D**). This indicates that these two chromosome ends were the most likely candidates for harboring integrated HHV6A in the ciHHV-6A and iciHHV-6A cell models, respectively. We confirmed that these chromosomal regions harbor the integrated virus by FISH using probes specific for the virus genome and the respective human chromosomes (**Figure 3E**). The integrated HHV-6A genome indeed was present in the respective chromosomal loci, highlighting that the 4C-seq analysis is an unbiased method to identify HHV-6 chromosomal integration sites.

### 4C-Seq Integration Site Analysis Using Nanopore Sequencing

**Figure 4:**
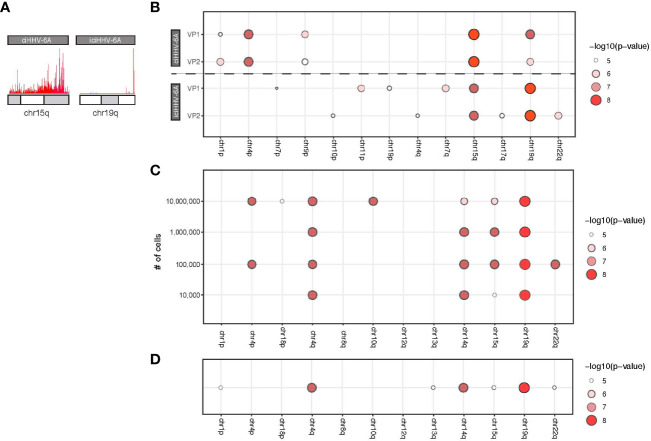
MinION-based 4C-seq human herpesvirus 6A (HHV-6A) integration analysis. **(A)** Karyotype plots showing 4C-seq MinION read mapping density at chr15q and chr19q for ciHHV-6A and iciHHV-6A cell models. **(B)** Chromosomal end integration site analysis using 4C-seq with MinION reads. As in **Figure 3D**, a statistical measure of the likelihood that a specific chromosome harbors integrated HHV-6A. Plotted are only the top scoring chromosome ends. **(C)** A titration series of cell dilutions was used for 4C-seq MinION analysis and was scored with statistical testing to identify potential HHV-6A integration chromosome ends. **(D)** MinION 4C-seq analysis results of a frozen iciHHV-6B+ human lymphocyte sample.

Next generation sequencing technologies have made it possible to generate large quantities of sequence data required for a variety of genomic assays including 4C-seq. However, 4C-seq library sizes derived from inverse PCR are relatively large and often difficult to cluster on Illumina flowcells. Long read sequencing platforms, including the Oxford Nanopore Technologies (ONT) minION, have become common an option for long read sequencing and have been applied for structural genome mapping approaches (De Coster et al., 2019). We therefore evaluated minION sequencing on the 4C-seq libraries as a method to identify integration sites from HHV-6 samples. We sequenced the same 4C-seq libraries with minION flowcells and obtained 150,646,076 bp of demuxable reads with a mean read length of 604.0 bp for the ciHHV-6A and iciHHV-6A sequencing run. For the cell counts titration sequencing run we obtained 419,121,238 reads with a mean read length of 730.4 bp. Alignment of reads that pass quality filters to the human genome reveals a similar grouping of telomere proximal reads at 15q and 19q for ciHHV-6A and iciHHV-6A samples, respectively (**Figure 4A**). Current 4C-seq peak calling software are not well-suited for long-read with low quality scores. We thus adapted our own method based off clustering of adjacent 4C-seq reads (methods). Using this approach, we found an overall comparable number of *trans* peaks called between replicates (**Figure 4B**). Thus, minION sequencing data represents a sequencing method to generate 4C-seq read data.

We applied the minION 4C-seq reads to investigate the required input material required for identifying HHV-6A integration sites by titrating the number of cells for 4C library construction. We compared a range of input cell quantities for 4C library construction (10^4^ to 10^7^ cells) using the iciHHV-6A cells combined with minION sequencing (**Figure 4C**). These results confirm that 19q harbors the integrated HHV-6A genome for iciHHV-6A SMC cells, and this can be reliably detected in as few as 100,000 cells for the 4C-seq assay. However, using just 10,000 cells 19q also scores highest, however there appears to be some ambiguity as 14q also scores highly. Finally, we also applied minION 4C-seq to detect HHV-6A integration sites using frozen patient-derived B cells from iciHHV-6A+ individuals for this assay. Like the iciHHV-6A SMC samples, 19q also scores as the likely integration site for HHV-6 integration in additional patient-derived lymphocyte samples (**Figure 4D**). FISH validation using chromosome 19q probes of the patient samples confirms HHV-6A integration into chromosome 19q (Aswad et al., 2020; Wight et al., 2020). Thus, 4C-seq libraries can be generated as little as 10,000 cells and using cryopreserved lymphocyte cell samples. In summary, this workflow allowed us to efficiently identify the chromosomal ends that most likely harbor the integrated virus genome.

## Discussion

HHV-6A and HHV-6B, potentially along with HHV-7 (Prusty et al., 2017) are the only known pathogens to integrate into human telomeres (Gulve et al., 2017; Prusty et al., 2017). While the chromatin mechanisms that govern HHV-6A latency and reactivation remain overall poorly characterized, it has been suggested that integration of the HHV-6A/B genome facilitates the maintenance of the virus genome latency (Arbuckle et al., 2010; Huang et al., 2014). The establishment quiescent/latent states has been investigated in a number of cell lines including 293T cells (Gravel et al., 2017). In these *in vitro*-generated cells and patient-derived iciHHV-6A cells, the virus genome transcriptionally silent (Saviola et al., 2019). These global RNA-sequencing data are in contrast to Kondo et al., who detected a few latency-associated transcripts by qRT-PCR (Kondo et al., 2002).

To investigate the establishment of a latent-like expression profile, we infected cells and performed RNA-sequencing during the first days of integration. Indeed, in early time points (days 1–3, **Figure 1**), we detect high expression levels of all HHV-6A RNA transcripts. However, over the period of 5 days post infection the viral transcripts were significantly reduced and became virtually undetectable by 7 days post infection. It remains unclear whether the RNA-expression is derived from and integrated or extrachromosomal virus genome. However, after 7 days post infection only integrated virus genome were detected when gene expression is absent.

Our prior analyses of the heterochromatin enrichment profiles of integrated HHV-6A genomes revealed that the integrated viral genome was resistant to MNase digestion, suggesting that the viral genome forms a compact chromatin domain (Saviola et al., 2019). Consistent with these findings, they found that the viral genome was enriched with the repressive posttranslational histone modifications H3K27me3 and H3K9me3. These results suggested that repressive chromatin structures likely play a role in viral gene silencing.

To further explore the chromatin-mediated mechanisms associated with silent chromatin, we applied the 4C-seq to determine the genome-wide chromatin contacts of HHV-6A based on nuclear proximity ligation. 4C-seq is designed to examine the genome for sequences contacting a selected genomic site of interest. Accordingly, we designed 4C-seq assays with viral viewpoint primers to identify the HHV-6 chromatin interactome. We generated high-resolution contact profiles for distinct viral viewpoints and detail the chromosomally integrated HHV-6 genomic organization. We find that clonally expanded *in vitro* HHV-6A (ciHHV-6A) cells display unique virus-host interactions relative to patient-derived iciHHV-6A cells. Overall, there are not many significantly detected inter-chromosomal or *trans* contacts across the human genome. However, the interactions that we detected were largely enriched with chromatin annotated as being quiescent or heterochromatin. Overall, this analysis indicates that the HHV-6A genome folds in a manner that it is capable of physically interacting with repressive chromatin and may explain some of the mechanisms used to silence virus gene expression in chromosomally integrated HHV-6A cells. To our knowledge, these results were the first reported chromatin interaction for a betaherpesvirus, and it is possible that these interactions between the virus and repressive chromatin elements play distinctive functions in HHV-6A latency.

We also found that the integrated HHV-6A genome forms several shared intra-chromosomal or *cis* contacts between the two ciHHV-6A cell types and that the virus genome exists in a highly folded state. It is possible that the HHV-6 genome forms a topologically associated domain within the virus and neighboring human sub-telomeric regions. Topologically associated domains or TADs are large regions of local intrachromosomal interactions. In support of viral TAD formation, we find a strong enrichment of telomeric sequence content in HHV-6A 4C-seq data. Interestingly, these domains appear to extend past the non-unique subtelomeric regions into the unique portion of the human genome and aligned reads cluster to the ends of 15q and 19q stand out in this data. Importantly, these regions score as the strongest interaction sites and confirm as being the integration chromosomes in independent FISH assays using probes to both virus and the identified chromosomal regions. Further experimental and computational methods including HiC and TAD calling algorithms are required to comprehensively determine HHV-6A TAD structures.

The identified *trans* interacting human sites as well as the strong signals along the distal ends of distinctive human chromosomes indicated that the 4C assay may prove useful to identify integration sites. Current 4C-seq peak calling methods are designed to identify interacting peaks based on monotonic shape as well as proximity to viewpoint regions. In addition, most 4C-seq peak calling methods are not designed to detect *trans* interactions between two unrelated genomes, although recent methodological improvements have been reported in the case of Epstein Barr Virus (Kim et al., 2020) and we were able to successfully apply such a method to our Illumina sequencing data.

To further establish 4C assays for calling integration sites, we developed a simple scoring method that scores and statistically evaluates candidate chromosomal integration sites. This method bins read alignments that occur at the distal 500 kb ends of each chromosome and performs statistical analysis to provide a useful score to evaluate chromosomal integration. For the ciHHV-6A and iciHHV-6A samples, this method accurately predicts the integration site. We further used this method to evaluate reads derived from a minION flow cell from a titration experiment with single replicates for each titration level. In each case, this method accurately identified validated integration regions as top scoring candidate regions and helped to demonstrate that as few as 10,000 – 100,000 cells can be used as input for 4C-seq assays.

Chromatin conformation capture methods have been previously applied to the study of physical chromatin interactions between host and viral genomes. For herpesvirus latency, HiC was used to demonstrate that the latent EBV will disassociate from repressive heterochromatin compartments and form new associations with transcriptionally permissive euchromatin upon reactivation (Moquin et al., 2018). 4C-seq was recently used with Burkitt’s lymphoma cells, showing that the latent EBV episomes make contact with transcriptionally repressive H3K9me3 sites as well as attachment sites associated with transcriptionally silent genes (Kim et al., 2020). Finally, 4C-seq has been applied to study the chromatin structures of the latent HIV proviral genome (Dieudonne et al., 2009).

Chromatin conformation capture libraries generally require a large amount of starting material, i.e. as many as 10 million cells (Nilsen, 2014). To enable the 4C assay toward a routine and throughput method to identify integration sites in clinically relevant samples, we performed the assay using a titration of cell numbers ranging from 10^4^ to 10^7^ cells. Indeed, our titration results demonstrate that we can reliably detect sites as low as 10,000 cells (**Figure 4**). However, with this low number of cells we identified a few potential spurious hits including chromosome 14. Another adaptation to the assay, is the use of the Nanopore sequencing platform. This newer sequencing platform has several advantages over Illumina, including cost and speed of data generation. In particular relevance to this study, 4C libraries generated *via* inverse PCR can be greater than 1 kb in size. The resulting Illumina libraries can be difficult to cluster, resulting in a poor output from the sequencing run at a higher cost. We therefore used Nanopore to generate data for integration analysis and find that using our scoring method that this is adequate for integration analysis. Due to the reduced read quality and relatively lower output, it remains to be determined if Nanopore-derived 4C-seq analysis compares to Illumina analysis for interaction peak analysis.

Finally, it is important to note that the resolution of 4C-seq can be as high as 1-2 Mbp for 4C-seq interactions (Krijger et al., 2020) and the annotation of the corresponding physical interactions can be challenging. Higher resolution 3C methods, including capture HiC can be applied in future studies to better study the nature of these identified physical interactions. These issues of scale are particularly important in the case of HHV-6A integration because the interactions between the virus and the host genomes are akin to *cis-trans* interactions. The HHV-6A genome is simultaneously acting like an independent chromosome (*trans*) that integrates into a particular human chromosome (*cis*) and we need to consider these interactions as a special case of 4C interactions, i.e., something like a “*cis-trans*” interaction. Because of this simultaneous “*cis*” nature, we chose to focus on the *trans* interactions in the 1–2 Mb range that is typical of the size of *cis* interactions reported in literature. Future studies aimed at more precisely defining the cellular factors and human contact points made by integrated HHV-6 will facilitate a more mechanistic understanding of viral gene silencing.

In summary, we have utilized a 4C-seq framework to identify both *trans* (virus-host) and *cis* (virus-virus) interactions that are formed within the human host cell nucleus. We further utilized this assay toward the unbiased identification of HHV-6A integration sites in human chromosomes. This optimally complements our recently developed optical mapping approach for the integrated HHV-6 genome (Wight et al., 2020). While research into the mechanisms and factors governing HHV-6A integration is an active area of ongoing research (Aimola et al., 2020), implementation of the methodology presented in this study provides important information on the silencing of the integrated virus genome and lays the foundation for high throughput detection of HHV-6A/B integration sites in clinical samples.

## Data Availability Statement

The datasets presented in this study can be found in online repositories. The names of the repository/repositories and accession number(s) can be found below: GSE162821.

## Ethics Statement

Specimens were obtained from the Fred Hutch Research Cell Bank, which prospectively collects and cryopreserves peripheral blood mononuclear cells (PBMCs) from donors and recipients. The University of Washington Institutional Review Board approved use of the iciHHV-6 specimens from the Fred Hutchinson Cancer Research Center and use of anonymized excess HHV-6-positive samples submitted for testing at the University of Washington Virology lab.

## Author Contributions

BK and SF designed and supervised the study and acquired funding. CZ and GA performed cell culture, qPCR, FISH, and flow cytometry experiments. MM, EH, PR, AR, and DG performed 4C-seq and RNA-seq experiments. MM and SF performed the analysis of the sequencing data. LF generated and provided cells. MM, AD, and SF wrote the manuscript. MM, BK, SF, LF, and GA revised the manuscript. All authors contributed to the article and approved the submitted version.

## Funding

This research was supported by the NIH R21AI121528, U54GM115516, and European Research Council grant number Stg 677673. 

## Conflict of Interest

The authors declare that the research was conducted in the absence of any commercial or financial relationships that could be construed as a potential conflict of interest.

## Footnotes

1
https://github.com/FrietzeLabUVM/4c_primer

2
https://egg2.wustl.edu/roadmap/web_portal/imputed.html#chr_imp

## Supplementary Material

Supplementary Figure 1 HHV-6A 4C-seq reads are enriched with telomeric repeats. Telomere content comparison was assessed by TelomereHunter (Feuerbach et al., 2019). Illumina data was simulated using ART (Huang et al., 2012). Reads were produced to cover the entire UCSC hg38 reference genome at 1X coverage. Then, several simulated reads equivalent to the number of reads sequenced between ciHHV-6A 4C-seq data were randomly sampled from the simulated data. TelomereHunter was run on all samples and a non-parametric Wilcoxon test was performed to compare 4C-seq reads to simulated reads (p-value = 0.002).

Supplementary Figure 2 HHV-6A *trans* interaction regions are enriched with repressive histone modifications H3K9me3 and H3K27me3. **(A)**. Genome tracks across chromosome 15 for ciHHV-6A showing significant 4C *trans* peak regions (top track), 4C signal coverage, HEK293T H3K9me3 ChIP signal and HEK293T H3K27me3 ChIP signal (bottom track). **(B)**. The same as **A**, but across chromosome 19 for iciHHV-6A samples.

Supplementary Figure 3 Similarities in chromatin state annotation in tissues related to HEK293 cells. **(A)**. The significant *trans* regions returned by the 4C window method were annotated using the 127 epigenomes chromHMM 25-state model as in **Figure 2D**. **(B, C)**. Annotations for fetal kidney and fetal adrenal gland tissues were then added for comparison with **A**.
