# Supplementary figures and images for: Higher-Order Chromatin Structures of Chromosomally Integrated HHV-6A Predict Integration Sites
Source: Front Cell Infect Microbiol. 2021 Feb 26;11:612656. doi: 10.3389/fcimb.2021.612656 (PMC7953476; doi:10.3389/fcimb.2021.612656)

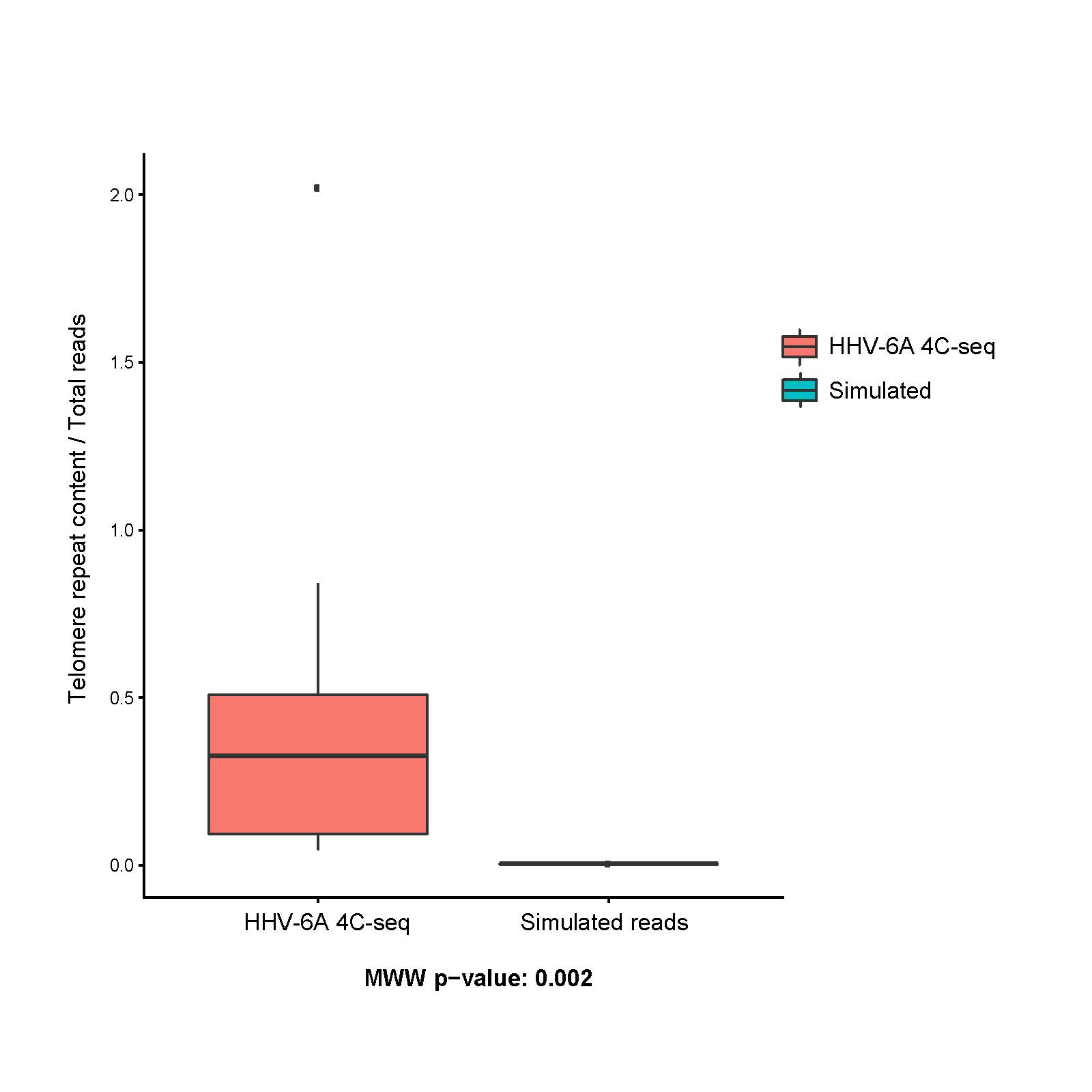

Supplement: Supplementary Figure 1 — HHV-6A 4C-seq reads are enriched with telomeric repeats. Telomere content comparison was assessed by TelomereHunter (Feuerbach et al., 2019). Illumina data was simulated using ART (Huang et al., 2012). Reads were produced to cover the entire UCSC hg38 reference genome at 1X coverage. Then, several simulated reads equivalent to the number of reads sequenced between ciHHV-6A 4C-seq data were randomly sampled from the simulated data. TelomereHunter was run on all samples and a non-parametric Wilcoxon test was performed to compare 4C-seq reads to simulated reads (p-value = 0.002). [file Image_1.jpeg]

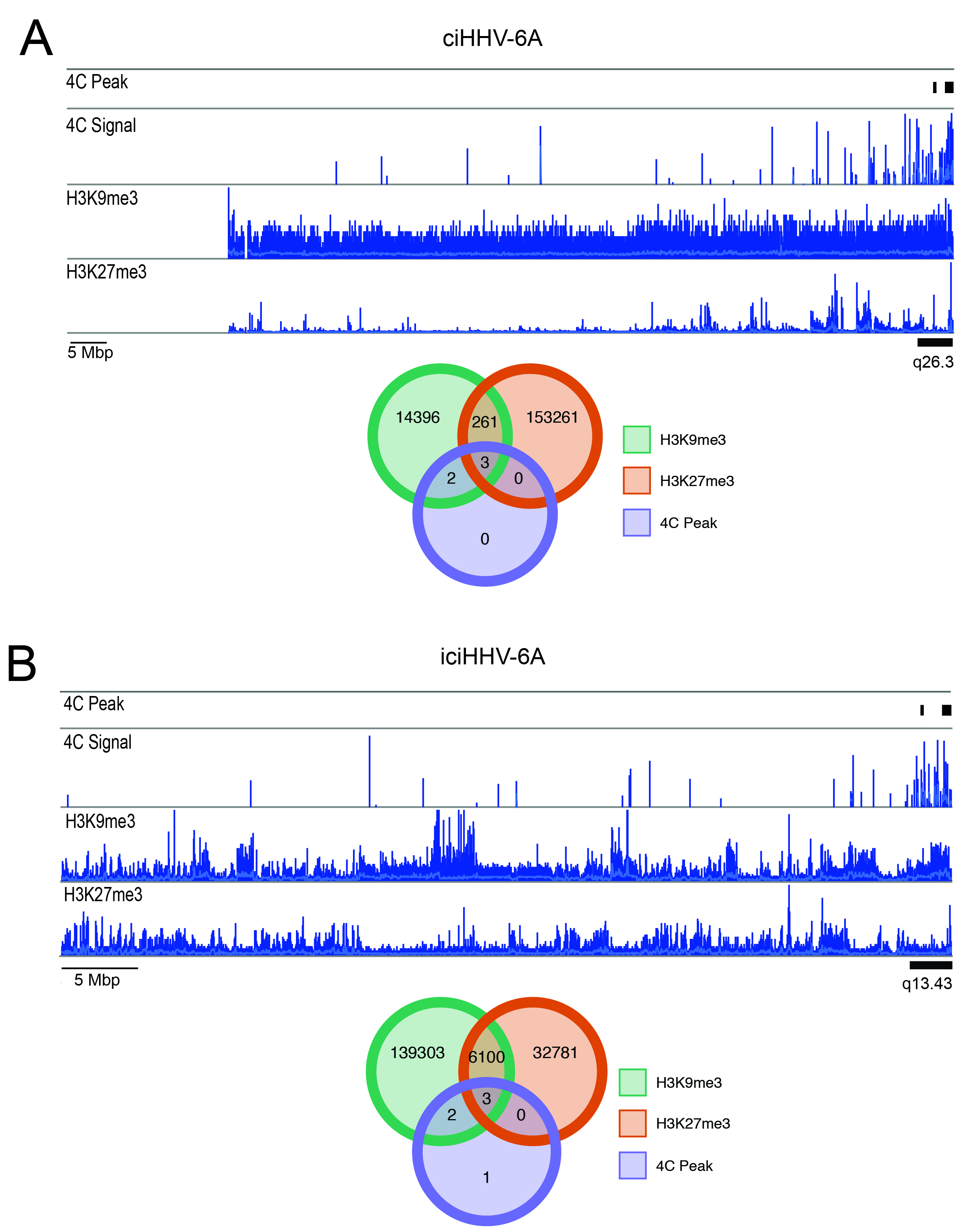

Supplement: Supplementary Figure 2 — HHV-6A trans interaction regions are enriched with repressive histone modifications H3K9me3 and H3K27me3. (A). Genome tracks across chromosome 15 for ciHHV-6A showing significant 4C trans peak regions (top track), 4C signal coverage, HEK293T H3K9me3 ChIP signal and HEK293T H3K27me3 ChIP signal (bottom track). (B). The same as A, but across chromosome 19 for iciHHV-6A samples. [file Image_2.jpeg]

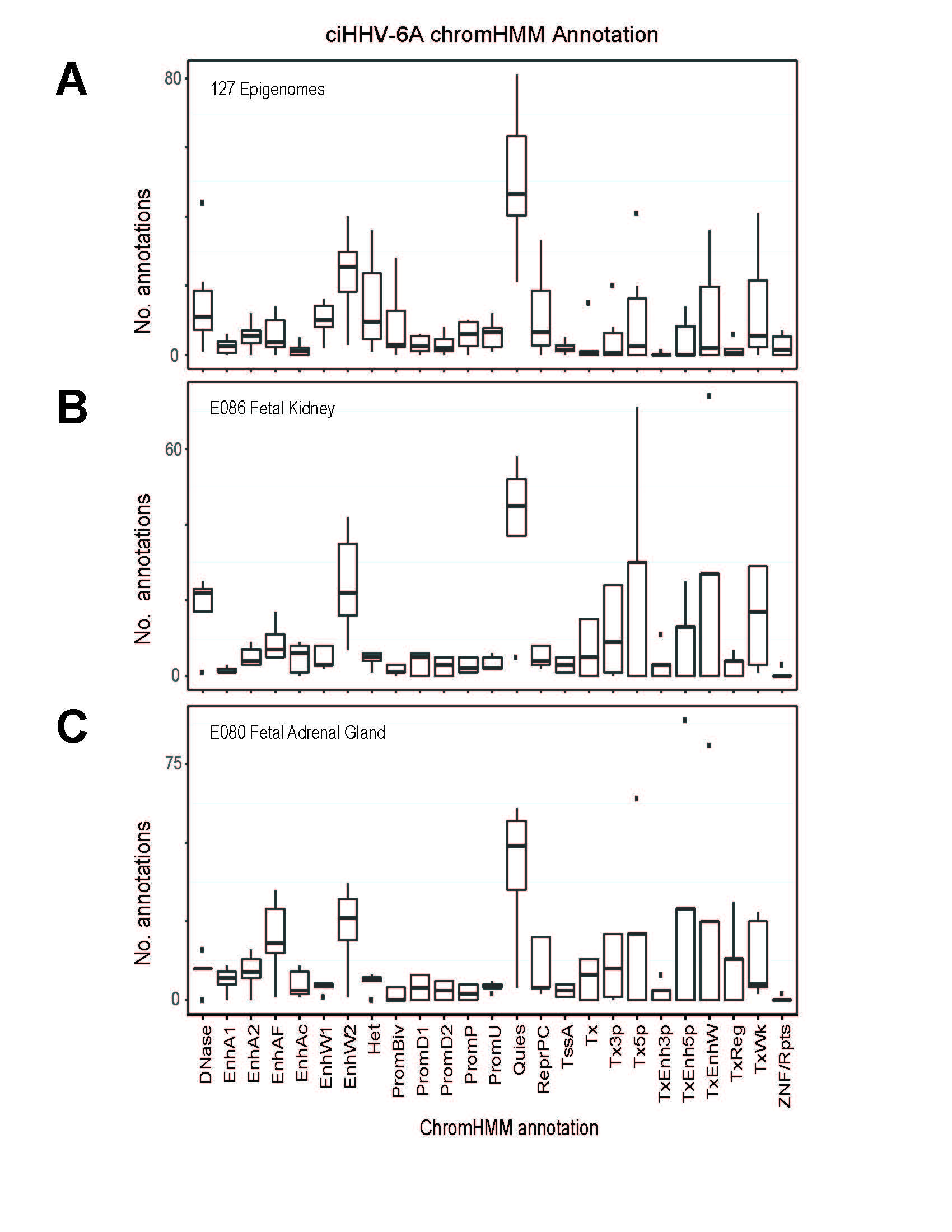

Supplement: Supplementary Figure 3 — Similarities in chromatin state annotation in tissues related to HEK293 cells. (A). The significant trans regions returned by the 4C window method were annotated using the 127 epigenomes chromHMM 25-state model as in Figure 2D . (B, C). Annotations for fetal kidney and fetal adrenal gland tissues were then added for comparison with A. [file Image_3.jpeg]
