# Supplementary material for: Higher-Order Chromatin Structures of Chromosomally Integrated HHV-6A Predict Integration Sites
Source: Front Cell Infect Microbiol. 2021 Feb 26;11:612656. doi: 10.3389/fcimb.2021.612656 (PMC7953476; doi:10.3389/fcimb.2021.612656)
Supplement: Supplementary file 4 [file Table_1.docx]

Supplemental Table 1: Primers used for 4C-seq

| **Primer** | **Primer sequence** | **Notes** |
| --- | --- | --- |
| VP1_F | TCGTCGGCAGCGTCAGATGTGTATAAGAGACAGACTTAGGGTACATAAAGCTT | VP1 Forward Primer |
| VP1_R | GTCTCGTGGGCTCGGAGATGTGTATAAGAGACAGCCACAAAACCCCATTATCTC | VP1 Reverse Primer |
| VP2_F | TCGTCGGCAGCGTCAGATGTGTATAAGAGACAGCCCACCCTGACATAAAGCTT | VP2 Forward Primer |
| VP2_R | GTCTCGTGGGCTCGGAGATGTGTATAAGAGACAGAAGAAACAGTAAATCTCTCGGT | VP2 Reverse Primer |
